# Development and validation of a model to predict cardiovascular death, nonfatal myocardial infarction, or nonfatal stroke in patients with type 2 diabetes mellitus and established atherosclerotic cardiovascular disease

**DOI:** 10.1186/s12933-022-01603-8

**Published:** 2022-08-27

**Authors:** Susanna R. Stevens, Matthew W. Segar, Ambarish Pandey, Yuliya Lokhnygina, Jennifer B. Green, Darren K. McGuire, Eberhard Standl, Eric D. Peterson, Rury R. Holman

**Affiliations:** 1 Duke Clinical Research Institute, Duke University School of Medicine, P.O. Box 17969, Durham, NC 27715 USA; 2 Division of Cardiology, University of Texas Southwestern Medical Center and Parkland Health and Hospital System, Dallas, TX USA; 3 Diabetes Research Group e.V. at Munich Helmholtz Center, Munich, Germany; 4 Diabetes Trials Unit, Radcliffe Department of Medicine, University of Oxford, Oxford, UK; 5 Present Address: Division of Cardiology, University of Texas Southwestern Medical Center, Dallas, TX USA; 6 Department of Cardiology, Texas Heart Institute, Houston, TX USA

**Keywords:** Atherosclerotic cardiovascular disease, Type 2 diabetes mellitus, Major adverse cardiovascular events, Risk modeling

## Abstract

**Background:**

Among individuals with atherosclerotic cardiovascular disease (ASCVD), type 2 diabetes mellitus (T2DM) is common and confers increased risk for morbidity and mortality. Differentiating risk is key to optimize efficiency of treatment selection. Our objective was to develop and validate a model to predict risk of major adverse cardiovascular events (MACE) comprising the first event of cardiovascular death, myocardial infarction (MI), or stroke for individuals with both T2DM and ASCVD.

**Methods:**

Using data from the Trial Evaluating Cardiovascular Outcomes with Sitagliptin (TECOS), we used Cox proportional hazards models to predict MACE among participants with T2DM and ASCVD. All baseline covariates collected in the trial were considered for inclusion, although some were excluded immediately because of large missingness or collinearity. A full model was developed using stepwise selection in each of 25 imputed datasets, and comprised candidate variables selected in 20 of the 25 datasets. A parsimonious model with a maximum of 10 degrees of freedom was created using Cox models with least absolute shrinkage and selection operator (LASSO), where the adjusted R-square was used as criterion for selection. The model was then externally validated among a cohort of participants with similar criteria in the ACCORD (Action to Control Cardiovascular Risk in Diabetes) trial. Discrimination of both models was assessed using Harrell’s C-index and model calibration by the Greenwood-Nam-D’Agostino statistic based on 4-year event rates.

**Results:**

Overall, 1491 (10.2%) of 14,671 participants in TECOS and 130 (9.3%) in the ACCORD validation cohort (n = 1404) had MACE over 3 years’ median follow-up. The final model included 9 characteristics (prior stroke, age, chronic kidney disease, prior MI, sex, heart failure, insulin use, atrial fibrillation, and microvascular complications). The model had moderate discrimination in both the internal and external validation samples (C-index = 0.65 and 0.61, respectively). The model was well calibrated across the risk spectrum—from a cumulative MACE rate of 6% at 4 years in the lowest risk quintile to 26% in the highest risk quintile.

**Conclusion:**

Among patients with T2DM and prevalent ASCVD, this 9-factor risk model can quantify the risk of future ASCVD complications and inform decision making for treatments and intensity.

**Supplementary Information:**

The online version contains supplementary material available at 10.1186/s12933-022-01603-8.

## Introduction

Among individuals with atherosclerotic cardiovascular disease (ASCVD), type 2 diabetes mellitus (T2DM) is common and confers increased risk of morbidity and mortality [1, 2]. Similarly, patients with T2DM are nearly three times as likely to develop major adverse cardiovascular events (MACE), defined as cardiovascular (CV) death, myocardial infarction (MI), and stroke, compared with those without diabetes [3, 4]. Consequently, prevention of MACE has been a major focus of clinical trials, therapeutic strategies and clinical practice guidelines for patients with T2DM.

Therapeutic decisions among these patients are ideally driven by the underlying risk of patients for CV complications. Yet, to date, models for this risk prediction are lacking, and those available have suboptimal performance and are outdated. The UKPDS Risk Engine has demonstrated reasonable accuracy to predict the risk of ASCVD events in patients with T2DM, yet this was developed among those free of baseline heart disease or stroke and at the time of T2DM diagnosis [5]. In contrast, there has been comparatively less attention directed toward predicting CV risk in individuals with both T2DM and established ASCVD [6]. An updated UKPDS outcomes model was developed to predict secondary MI and stroke events over a median duration of 17 years [7], but the risk model was developed using simulation studies, was not externally validated, and aimed to predict risk over a long-term, near 20-year follow-up. Given that individuals with ASCVD are more than twice as likely to have MACE compared with primary prevention subgroups within 4 years, risk stratification of patients is important to inform treatment decisions [6, 8].

In this study, our aim was to develop a model to predict intermediate-term ASCVD events among individuals with both T2DM and ASCVD enrolled in the Trial Evaluating Cardiovascular Outcomes with Sitagliptin (TECOS) and externally validate the findings in participants in the ACCORD (Action to Control Cardiovascular Risk in Diabetes) trial.

## Methods

Because of the sensitive nature of the data collected for this study, requests to access the dataset from qualified researchers trained in human subject confidentiality protocols may be submitted at dcri.org/data-sharing.

### Study design and participants

TECOS (NCT00790205) was a double-blind, multinational, placebo-controlled trial evaluating the CV safety of adding sitagliptin to usual care in patients with T2DM and established ASCVD. Details of the trial design and primary results, as well as the study protocol and other supplementary trial resources, have been published [9, 10]. Briefly, the intention-to-treat population comprised 14,671 participants from 38 countries (662 sites) who were enrolled between December 2008 and July 2012 and followed for a median of 3.0 years. For inclusion, patients were ≥ 50 years old with T2DM, ASCVD, and HbA_1c_ values of 6.5–8.0%. Participants were taking insulin or oral antihyperglycemic agents at baseline with the exception of dipeptidyl peptidase-4 inhibitors, glucagon-like peptide 1 (GLP-1) receptor agonists, and rosiglitazone. Participants were excluded if they had two or more severe hypoglycemic events in the last 12 months or their baseline estimated glomerular filtration rate (eGFR) was < 30 mL/min/1.73 m^2^. Participants were randomized 1:1 to treatment with sitagliptin or placebo with doses based on eGFR values. Concomitant medications could be adjusted during the study according to local standards of care. The trial was run jointly by the Duke Clinical Research Institute and the University of Oxford Diabetes Trials Unit in an academically independent collaboration with the sponsor, Merck Sharp and Dohme. All participants provided written informed consent, and the protocol was approved by the ethics committees at each site.

### Outcome of interest

The clinical outcome for the present analyses is three-point composite MACE, defined as the time to first nonfatal MI, nonfatal stroke or CV death. Events were adjudicated by an independent clinical events committee without knowledge of treatment assignment [9].

### Candidate variables

In total, 59 baseline variables were considered as candidates for model development (see Additional file 1: Supplemental Methods). Summary statistics for these variables are shown in Table 1, with the amount of nonmissing data shown in Additional file 1: Table S1. After evaluating collinearity and missingness, models were developed using 33 baseline variables. A detailed description of how the variables that were ultimately considered as candidates were chosen is provided in the Supplemental Material. In brief, variables encompassed a range of domains including demographic, anthropometric measures, medical history, laboratory values, current medication use, and diabetes complications. Variables with > 25% missingness were excluded, and the remaining variables with missing data were imputed. A single microvascular diabetic complication variable was used as a composite of blindness, amputation, foot ulcer, diabetic neuropathy or retinopathy.

**Table 1:** Baseline patient characteristics in the overall cohort and stratified by major adverse cardiovascular event (MACE)

	Overall (N = 14,671)	No MACE (N = 13,180)	MACE (N = 1491)	p*
Age, y	65 (60, 71)	65 (59, 71)	68 (62, 73)	< 0.0001
Female sex	4297 (29.3%)	3926 (29.8%)	371 (24.9%)	0.0002
Race				< 0.0001
White	9957 (67.9%)	8896 (67.5%)	1061 (71.2%)	
Black	447 (3.0%)	395 (3.0%)	52 (3.5%)	
Asian	3265 (22.3%)	2995 (22.7%)	270 (18.1%)	
Other	1002 (6.8%)	894 (6.8%)	108 (7.2%)	
Hispanic ethnicity	1798 (12.3%)	1645 (12.5%)	153 (10.3%)	0.2128
Region				0.0837
Latin America	1471 (10.0%)	1348 (10.2%)	123 (8.2%)	
Asia Pacific and Other	4565 (31.1%)	4120 (31.3%)	445 (29.8%)	
Western Europe	2076 (14.2%)	1876 (14.2%)	200 (13.4%)	
Eastern Europe	3965 (27.0%)	3548 (26.9%)	417 (28.0%)	
North America	2594 (17.7%)	2288 (17.4%)	306 (20.5%)	
Duration of type 2 diabetes, y	10 (5, 16)	10 (5, 16)	11 (6, 17)	< 0.0001
HbA1c, %	7.2 (6.8, 7.6)	7.2 (6.8, 7.6)	7.2 (6.8, 7.7)	0.0095
Body mass index, kg/m^2†^	29.5 (26.3, 33.3)	29.5 (26.3, 33.3)	29.5 (26.3, 33.3)	0.0228
Systolic blood pressure, mmHg	134 (124, 145)	133 (124, 145)	135 (124, 147)	0.0024
Diastolic blood pressure, mmHg^†^	79 (70, 84)	79 (70, 84)	78 (70, 85)	< 0.0001
Heart rate, bpm†	72 (65, 79)	72 (64, 79)	72 (65, 80)	0.0002
eGFR, mL/min/1.73 m^2†^	73 (60, 88)	73 (60, 88)	67 (55, 84)	< 0.0001
Chronic kidney disease, eGFR < 60 mL/min/1.73 m^2^	3324 (22.9%)	2825 (21.6%)	499 (33.9%)	< 0.0001
UACR, mg/g	10.5 (3.5, 33.6)	10.0 (3.5, 30.9)	16.9 (5.3, 62.5)	< 0.0001
Hemoglobin, g/L^†^	137 (127, 147)	137 (127, 147)	136 (125, 147)	0.0007
Non-HDL-c, mg/dL	114 (92, 144)	113 (91, 143)	117 (94, 149)	0.0012
HDL-c, mg/dL^†^	42 (35, 50)	42 (35, 50)	41 (34, 48)	0.0003
LDL-c, mg/dL	84 (65, 109)	83 (65, 108)	86 (66, 113)	< 0.0001
Triglycerides, mg/dL	142 (103, 199)	142 (103, 199)	144 (103, 204)	0.6998
Prior myocardial infarction	6255 (42.6%)	5500 (41.7%)	755 (50.6%)	< 0.0001
≥ 50% coronary stenosis	7687 (52.4%)	6847 (51.9%)	840 (56.3%)	0.0314
Prior PCI	5714 (39.5%)	5143 (39.6%)	571 (38.8%)	0.3501
Prior CABG	3664 (25.0%)	3253 (24.7%)	411 (27.6%)	0.0204
Prior stroke	2555 (17.4%)	2201 (16.7%)	354 (23.7%)	< 0.0001
Prior TIA	566 (3.9%)	492 (3.7%)	74 (5.0%)	0.0121
≥ 50% stenosis in the carotid artery	860 (5.9%)	750 (5.7%)	110 (7.4%)	0.0084
Peripheral arterial disease	2433 (16.6%)	2203 (16.7%)	230 (15.4%)	0.3483
NYHA class				< 0.0001
No CHF	12,028 (84.4%)	10,935 (85.2%)	1093 (77.1%)	
I	535 (3.8%)	468 (3.6%)	67 (4.7%)	
II	1312 (9.2%)	1126 (8.8%)	186 (13.1%)	
III	360 (2.5%)	295 (2.3%)	65 (4.6%)	
IV	13 (0.1%)	6 (< 0.1%)	7 (0.5%)	
Cigarette smoking status				0.0047
Current	1678 (11.4%)	1481 (11.2%)	197 (13.2%)	
Former	5844 (39.8%)	5228 (39.7%)	616 (41.3%)	
Never	7149 (48.7%)	6471 (49.1%)	678 (45.5%)	
Hypertension	12,648 (86.2%)	11,318 (85.9%)	1330 (89.2%)	0.0004
Dyslipidemia	11,240 (76.6%)	10,096 (76.6%)	1144 (76.7%)	0.8544
COPD	1117 (7.6%)	955 (7.2%)	162 (10.9%)	< 0.0001
Atrial fibrillation/flutter	1167 (8.0%)	963 (7.3%)	204 (13.7%)	< 0.0001
Cancer within the past 5 years	327 (2.2%)	286 (2.2%)	41 (2.7%)	0.1239
Depression	1172 (8.0%)	1029 (7.8%)	143 (9.6%)	0.0135
Liver disease	273 (1.9%)	250 (1.9%)	23 (1.5%)	0.4512
Any microvascular complication	4608 (31.4%)	4040 (30.7%)	568 (38.1%)	< 0.0001
Blindness	235 (1.6%)	205 (1.6%)	30 (2.0%)	0.1751
Retinopathy	1864 (12.7%)	1616 (12.3%)	248 (16.6%)	< 0.0001
Amputation	377 (2.6%)	315 (2.4%)	62 (4.2%)	< 0.0001
Diabetic neuropathy	3354 (22.9%)	2938 (22.3%)	416 (27.9%)	< 0.0001
Foot ulcers	393 (2.7%)	327 (2.5%)	66 (4.4%)	< 0.0001
Albuminuria				< 0.0001
Normal	9274 (79.7%)	8472 (80.7%)	802 (70.5%)	
Microalbuminuria	1924 (16.5%)	1664 (15.9%)	260 (22.9%)	
Macroalbuminuria	437 (3.8%)	362 (3.4%)	75 (6.6%)	
Insulin	3408 (23.2%)	2988 (22.7%)	420 (28.2%)	< 0.0001
Sulfonylurea	6645 (45.3%)	5962 (45.2%)	683 (45.8%)	0.2143
Metformin	11,966 (81.6%)	10,837 (82.2%)	1129 (75.7%)	< 0.0001
ACE inhibitor or ARB	11,555 (78.8%)	10,342 (78.5%)	1213 (81.4%)	0.0140
Beta blocker	9322 (63.5%)	8295 (62.9%)	1027 (68.9%)	< 0.0001
Calcium channel blocker	4961 (33.8%)	4427 (33.6%)	534 (35.8%)	0.0732
Diuretic	6020 (41.0%)	5247 (39.8%)	773 (51.8%)	< 0.0001
Aldosterone antagonist	839 (5.7%)	696 (5.3%)	143 (9.6%)	< 0.0001
Aspirin	11,518 (78.5%)	10,398 (78.9%)	1120 (75.1%)	< 0.0001
Thienopyridine	3187 (21.7%)	2845 (21.6%)	342 (22.9%)	0.4722
VKA	1000 (6.8%)	818 (6.2%)	182 (12.2%)	< 0.0001
NSAIDs	496 (3.4%)	446 (3.4%)	50 (3.4%)	0.9091
Statin	11,719 (79.9%)	10,591 (80.4%)	1128 (75.7%)	< 0.0001
Ezetimibe	761 (5.2%)	677 (5.1%)	84 (5.6%)	0.7233
Fibrate	943 (6.4%)	855 (6.5%)	88 (5.9%)	0.3220

MACE is a composite of first nonfatal myocardial infarction (MI), nonfatal stroke or cardiovascular death. Data shown are median (25th, 75th percentile) or n (%). Numbers nonmissing are shown separately in Additional file 1: Table S1. ACE: angiotensin-converting enzyme; ARB, angiotensin receptor blocker; CHF: congestive heart failure; COPD: chronic obstructive pulmonary disorder; eGFR: estimated glomerular filtration rate; HDL-c: high-density lipoprotein cholesterol; LDL-c: low-density lipoprotein cholesterol; NSAIDs: nonsteroidal anti-inflammatory drugs; PCI: percutaneous coronary intervention; TIA: transient ischemic attack; UACR: urine albumin-to-creatinine ratio; VKA: vitamin K antagonist

*P-values are calculated from univariable Cox proportional hazards regression models; multiple imputation was used when missing data were present

^†^When relationships between continuous variables and MACE are non-linear the p-value is for the model containing two piecewise linear splines

### Model development

Details on the model development are provided in the Additional file 1: Supplemental Materials. In sum, two Cox proportional hazards regression models for MACE were developed: (1) a “full” model that was not restricted in the number of variables; and (2) a parsimonious model intended to be easier to use as a risk prediction tool in clinical settings.

#### Variable selection and risk prediction

Twenty-five imputed datasets were created using SAS PROC MI with the method of fully conditional specification [11]. Variables with more than 25% missing were imputed but were not candidates for the final model. The linearity assumption of Cox regression was tested for each continuous variable, and the estimated association with the hazard of MACE was plotted using the model with restricted cubic splines. In the case of non-linearity, a cut-point was chosen based on visual inspection of the plots, and two linear splines were defined as candidates for model selection [12]. Variables selected in at least 20 of the 25 datasets were used in the final models [13]. The full model was developed using stepwise variable selection with alpha = 0.05 to enter and to stay in the model. The resulting model included all levels of categorical variables and both linear splines even when only some levels of the covariate were selected. The parsimonious model was fitted using Cox proportional hazards regression with least absolute shrinkage and selection operator (LASSO) methods [14]. A maximum of 10 degrees of freedom of only demographic and medical history variables was prespecified. The adjusted R-square statistic was used as criterion for selection.

#### Model evaluation

Cox proportional hazard ratios and p-values were calculated using Rubin’s method to combine the estimates from the 25 imputed datasets [13]. Discrimination of both models was assessed using Harrell’s C-index [15]. Finally, model calibration was assessed by the Greenwood-Nam-D’Agostino (GND) statistic [16] based on 4-year event rates. For model calibration and testing of modeling assumptions, only the first imputed dataset was used.

#### Statistical analysis

All variables that were considered for the predictive model were summarized overall and by presence of a MACE event. Continuous variables were summarized with median (25th, 75th percentile) and categorical variables with number and percentage. The numbers of nonmissing values before imputation are presented for each potential predictor. When relationships were found to be non-linear, the p-value corresponds to the model containing two piecewise linear splines. The cumulative incidence of MACE was displayed across quintiles of risk with differences assessed using the log-rank test. All analyses were performed using SAS version 9.4 and R version 3.6.3, with a p-value < 0.05 indicating significance.

### External validation of the risk score

The parsimonious model risk score was externally validated in a separate cohort of individuals with T2DM and prevalent ASCVD from the ACCORD trial [17, 18]. Briefly, ACCORD was a randomized, multicenter trial that evaluated whether intensive glycemic control (target HbA1c < 6%) versus standard control (target HbA1c 7–7.9%) reduced the risk of MACE. Recruitment occurred among 77 clinical centers in the United States and Canada in two phases, between January and June 2001 and from February 2003 through October 2005. The mean follow-up was 5.0 years. Participants were aged 40–79 years with T2DM and inadequate glycemic control (HbA1c ≥ 7.5%) and either established ASCVD or aged 55–79 years with left ventricular hypertrophy, albuminuria, atherosclerosis, or two or more other ASCVD risk factors (hyperlipidemia, hypertension, obesity or current smoking). Given that the results of the primary trial were null, patients randomized to both treatment arms were included. Similar to the derivation (TECOS) cohort, participants without ASCVD at baseline, age < 50 years, HbA1c > 8%, or eGFR < 30 were excluded. The primary outcome of interest was three-point composite MACE. Each of the components (nonfatal MI, nonfatal stroke, or CV death) were adjudicated outcomes of the ACCORD trial [17]. Model evaluation was assessed similar to the primary analysis. There were no missing data in the ACCORD cohort.

## Results

Baseline characteristics for the 14,671 TECOS participants in the overall cohort and stratified by first event of the MACE composite outcome are provided in Table 1. Overall, 1491 (10.2%) of the participants had a primary outcome event (MACE) at a rate of 3.6 events per 100 patient years of follow-up. The first event of the composite was CV death for 607, non-fatal MI for 578, non-fatal ischemic stroke for 283, nonfatal hemorrhagic stroke for 17, and nonfatal stroke of unknown type for 6. Participants who developed MACE were more commonly male, older, and had a higher frequency of prior MI, heart failure, atrial fibrillation, and chronic obstructive pulmonary disease (COPD). Participants who experienced MACE events also had lower eGFR and higher urine albumin-to-creatinine ratio levels at baseline.

### Development and internal validation of the full model

**Fig. 1:**
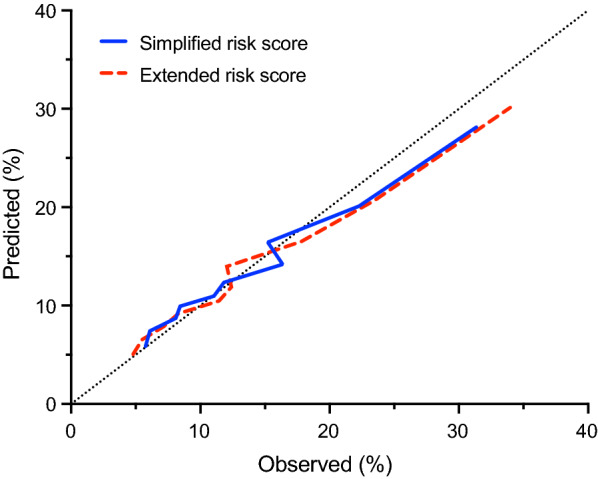
Predicted versus observed major adverse cardiovascular events (MACE) 4-year rates of the simplified and extended risk scores based on deciles of predicted risk. MACE is a composite of first nonfatal myocardial infarction, nonfatal stroke, or cardiovascular death

In multivariable Cox analysis of the full model, the identified variables included age, prior stroke, prior MI, eGFR, male sex, atrial flutter or fibrillation, insulin use, microvascular diabetic complications, heart failure, New York Heart Association (NYHA) class, non-high-density lipoprotein cholesterol, diastolic blood pressure, heart rate, albuminuria, coronary artery disease, body mass index < 25 kg/m^2^, current smoking, ≥ 50% carotid artery stenosis, dyslipidemia, and COPD (Table 2). In the internal validation cohort, the C-index for discrimination was 0.68 (95% CI 0.66–0.69). If hemorrhagic stroke were excluded as part of the composite endpoint, selected variables and the C-index would be unchanged (Additional file 1: Table S2). Calibration across deciles of predicted and observed risk is shown in Fig. 1 (GND chi-square = 7.6, p = 0.570).

**Table 2:** Multivariable adjusted model output parameters using variables in the extended risk score

Variable	Parameter estimate	HR (95% CI)	p
Age, per 10-year increase	0.311034	1.36 (1.27–1.47)	< 0.0001
Stroke	0.520675	1.68 (1.48–1.91)	< 0.0001
Myocardial infarction	0.382486	1.47 (1.31–1.63)	< 0.0001
eGFR			< 0.0001
HR for 10 unit increase to 80 mL/min/1.73 m^2^	− 0.157483	0.85 (0.82–0.89)	
HR for 10 unit increase above 80 mL/min/1.73 m^2^	0.069763	1.07 (1.02–1.13)	
Male	0.347975	1.42 (1.24–1.61)	< 0.0001
NYHA Class (No CHF is reference)			< 0.0001
I	0.151523	1.16 (0.91–1.49)	
II	0.352289	1.42 (1.22–1.66)	
III	0.395279	1.48 (1.15–1.92)	
IV	1.472099	4.36 (1.88–10.10)	
Non-HDL-c, HR for 10-unit increase in mg/dL	0.031708	1.03 (1.02–1.05)	< 0.0001
Insulin use	0.286234	1.33 (1.18–1.51)	< 0.0001
Diastolic blood pressure			< 0.0001
HR for 10 mmHg increase to 80	− 0.130286	0.88 (0.82–0.95)	
HR for 10 mmHg increase above 80	0.190161	1.21 (1.09–1.34)	
Heart rate			0.0001
HR for 10 bpm increase to 60	− 0.370442	0.69 (0.55–0.87)	
HR for 10 bpm increase above 60	0.104525	1.11 (1.05–1.17)	
Albuminuria (reference is none)			0.0002
Microalbuminuria	0.256595	1.29 (1.12–1.49)	
Macroalbuminuria	0.368159	1.45 (1.13–1.85)	
Coronary artery disease	0.205405	1.23 (1.10–1.38)	0.0004
Atrial flutter or fibrillation	0.283484	1.33 (1.13–1.55)	0.0004
Body mass index			0.0005
HR for 1 kg/m^2^ increase to 25 kg/m^2^	− 0.098754	0.91 (0.86–0.95)	
HR for 1 kg/m^2^ increase above 25 kg/m^2^	0.004252	1.00 (0.99–1.02)	
Smoking (reference is never)			0.0009
Current	0.290684	1.34 (1.13–1.58)	
Former	− 0.012079	0.99 (0.88–1.11)	
Any diabetes-specific microvascular comorbidity (blindness, amputation, foot ulcer, diabetic neuropathy, or retinopathy)	0.147577	1.16 (1.04–1.30)	0.0101
≥ 50% stenosis of carotid artery	0.243316	1.28 (1.05–1.55)	0.0152
Dyslipidemia	− 0.146350	0.86 (0.76–0.98)	0.0229
COPD	0.185822	1.20 (1.02–1.43)	0.0323

COPD: chronic obstructive pulmonary disorder; eGFR: estimated glomerular filtration rate; HDL-c: high-density lipoprotein cholesterol; NYHA: New York Heart Association

* The baseline survival function is 0.4610 at 1 year, 0.2211 at 2 years, 0.1024 at 3 years, and 0.0480 at 4 years

### Development and internal validation of the simplified model

**Fig. 2:**
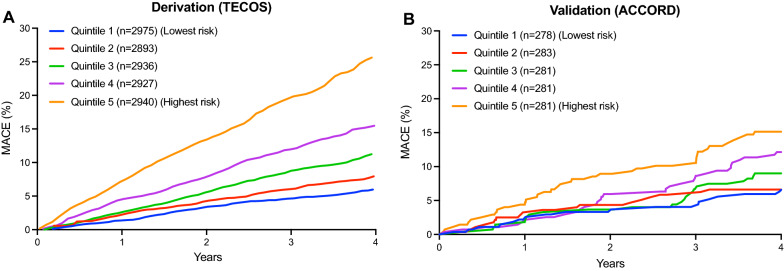
Cumulative incidence of MACE across quintiles of the risk score in the **A** derivation (TECOS) and **B** validation (ACCORD) cohorts. MACE is a composite of first nonfatal myocardial infarction, nonfatal stroke, or cardiovascular death

In multivariable Cox analysis of the parsimonious model, the identified variables were age, male sex, prior stroke, prior MI, chronic kidney disease (eGFR < 60), heart failure, atrial flutter or fibrillation, insulin use, and microvascular diabetic complications (Table 3). From the 9 identified covariates, we created a risk score for predicting MACE. The risk score demonstrated moderate discrimination with a C-index of 0.65 (95% CI 0.64–0.67) and acceptable calibration (GND chi-square = 11.4, p = 0.25) (Fig. 1). The cumulative incidence of MACE at 4 years increased in a graded fashion across quintiles of increasing modeled risk, from 6.0% in the lowest risk quintile to 25.8% in the highest risk quintile (log-rank p < 0.001; Fig. 2A). If the stroke portion of the composite were limited to ischemic and unknown type, model calibration and the variables selected for the simplified model would be similar (Additional file 1: Table S3).

**Table 3:** Multivariable adjusted model output parameters using variables in the parsimonious risk score

Variable	Parameter Estimate^*^	HR (95% CI)	p
Prior stroke	0.509145	1.66 (1.47–1.88)	< 0.0001
Age (per 10-year increase)	0.276077	1.32 (1.23–1.41)	< 0.0001
Chronic kidney disease	0.414291	1.51 (1.35–1.70)	< 0.0001
Prior myocardial infarction	0.377331	1.46 (1.31–1.62)	< 0.0001
Male	0.333232	1.40 (1.24–1.57)	< 0.0001
Heart failure	0.339297	1.40 (1.24–1.59)	< 0.0001
Insulin use	0.302525	1.35 (1.20–1.52)	< 0.0001
Atrial fibrillation or flutter	0.325465	1.38 (1.19–1.62)	< 0.0001
Any diabetes-specific microvascular comorbidity (blindness, amputation, foot ulcer, diabetic neuropathy, or retinopathy)	0.200567	1.22 (1.10–1.36)	0.0003

*The baseline survival function is 0.9976 at 1 year, 0.9953 at 2 years, 0.9929 at 3 years, and 0.9905 at 4 years

### External validation of the simplified model

Among 10,251 participants in the ACCORD trial, 6642 were excluded from the present analyses for non-ASCVD at baseline, 2119 for HbA1c > 8%, 72 for age < 50 years, and 14 for missing follow-up. The coefficients in Table 3 were used to calculate the risk score for each of the 1404 participants in the external validation cohort; 130 of the participants (9.3%) had MACE within 4 years of follow-up. The baseline characteristics of ACCORD participants are shown in Additional file 1: Table S4. The external validation cohort was of similar age and sex to TECOS but had a somewhat different disease profile. Both had T2DM and established ASCVD, but those in ACCORD had more prior MI, heart failure, coronary artery bypass grafting, carotid stenosis, higher HbA1c, more insulin use, and higher eGFR, whereas TECOS had higher atrial fibrillation, high-density lipoprotein cholesterol, diastolic blood pressure, heart rate, and chronic kidney disease. In discrimination analysis, the C-index of the model containing only the 9-variable risk score was 0.61 (95% CI 0.56–0.66). Further, no evidence of miscalibration was observed (GND chi-square = 9.31, p = 0.32). The cumulative incidence of MACE at 4 years increased in a graded fashion across risk-estimated quintiles, from 6.3% in quintile 1 to 15.2% in quintile 5 (log-rank p < 0.001; Fig. 2B).

## Discussion

In this study, a simple novel risk score was developed and validated, and shown to have reasonable accuracy in predicting MACE events (nonfatal MI, stroke, or CV death) among patients with T2DM and ASCVD. Several important findings were observed. First, the developed risk score incorporated commonly used clinical and laboratory variables, and could identify individuals with 4-year risk of MACE ranging from 6 to 25%. Second, while the more complex model of 19 variables resulted in better discrimination, a simplified risk score of only 9 patient characteristics retained much of the operating characteristics of the larger, more complex model. Finally, the score performed well in an external cohort of patients with T2DM and ASCVD. In all, the findings suggest a novel method to identify the highest risk patients and inform targeting of ASCVD secondary prevention strategies in patients with T2DM and prevalent ASCVD.

Management of patients with T2DM has historically been focused on the achievement of glycemic targets. However, newer classes of medications—such as sodium-glucose cotransporter 2 inhibitors (SGLT2i) and GLP-1 receptor agonists—have been shown to reduce the risk of CV events in patients with T2DM and established ASCVD [19–21]. The mechanisms by which these drugs achieve CV benefit and whether these benefits exists across the entire spectrum of ASCVD risk, though, are unclear. Additionally, these newer agents are associated with considerable healthcare costs—especially among low-income countries [22, 23]. Thus, tools to stratify patients with T2DM and ASCVD are needed to most efficiently use these medications, especially in resource-limited settings, and guide further therapy [24]. Similarly, tools to identify individuals most at risk for MACE may help healthcare providers meet quality care metrics and allocate resources to the highest risk patients.

Current American College of Cardiology Foundation/American Heart Association guidelines recommend ASCVD risk stratification in patients with T2DM using available global risk calculators [25]. Unfortunately, existing risk calculators have not been evaluated in individuals with T2DM and above-average ASCVD risk, or specifically with prevalent ASCVD as in the present analyses. Thus, risk stratification models to identify which patients may benefit from pharmacologic interventions, such as GLP-1 receptor agonists or SGLT-2 inhibitor therapy, could be beneficial to allocate resources and guide therapeutic decision making, especially relevant for proprietary therapies for which access and cost effectiveness are key considerations. Similarly, prediction models can help inform patients about their individual risk and prognosis [26]. Results from such risk scores can be used to educate and motivate patients, promoting physical activity and lifestyle modifications. The 9-variable risk prediction tool was developed to address this knowledge gap and predict MACE among individuals with prevalent ASCVD and T2DM.

The present findings also build on previous MACE risk prediction tools from individual cohorts, including the Framingham Heart Study, Swedish National Diabetes Register (NDR), and U.K. Prospective Diabetes Study (UKPDS) cohorts [27–30]. While the Framingham risk score includes participants with prevalent ASCVD, the risk score was not designed for individuals with T2DM and has been shown to have decreased accuracy in this population [31]. Conversely, the NDR risk score is designed for individuals free of baseline ASCVD and is not generalizable to individuals with a history of ASCVD. Finally, the UKPDS Risk Engine was developed using data collected between 1977 and 1991 from individuals with newly diagnosed T2DM aged 25–65, and may not reflect the current landscape of medical practices and burden of disease seen in individuals with long-standing T2DM, especially those with established ASCVD [5]. For example, the UKPDS score does not include current medications, such as insulin use, which is incorporated in our risk score and reflects current standard-of-care treatment regimens for those with advanced T2DM [32]. Similarly, the UKPDS Risk Engine was designed to predict long-term MACE risk (up to 20+ years). By contrast, our risk score derivation included a diverse cohort of Black and White men and women with above-average short-term ASCVD risk from a recent, clinical trial-based cohort to allow for robust and personalized 4-year estimates of MACE in patients with T2DM and prevalent ASCVD.

### Study strengths and limitations

The present study has several strengths, including derivation of the models in a large cohort of participants from the TECOS trial, analyses of data from patients with contemporary clinical care, a large number of events to analyze, use of advanced statistical techniques to identify and analyze variables, and validation of the models in an external cohort of patients with T2DM and ASCVD.

This study also has notable limitations. The TECOS trial was conducted between 2008 and 2012, and certain biomarkers associated with worsening CV outcomes, such as high-sensitivity troponin, natriuretic peptide, C-reactive peptide, and coronary calcium levels, were not available to assess their potential contributions to the models. However, these data are not routinely collected in individuals with stable T2DM and ASCVD. Similarly, the TECOS trial excluded individuals with severe kidney dysfunction—a known risk factor of worsening CV events. Time since prior ASCVD at baseline was not collected but could be potentially informative; however this could be variable in relation to the baseline examination of the trial. Although both populations had T2DM and ASCVD, the participant baseline comorbidities in TECOS and ACCORD occurred with different frequencies. Despite these differences, the model performed well in the validation cohort. Although defined similarly to other studies, in hindsight, the prespecified MACE endpoint for both TECOS and ACCORD studies would have been better if it did not include hemorrhagic stroke, since hemorrhagic stroke is thought to have a different risk profile than other components of the composite. However, if hemorrhagic stroke were removed from the endpoint, only 14 events (< 1%) would be lost and all predictors in the models would remain significant. Details for the models of CV death, nonfatal MI, or nonfatal ischemic stroke (without hemorrhagic stroke) are presented in Additional file 1: Tables S2 and S3. No measures of socioeconomic status (SES) were available to be considered for the model. Lower SES has been shown to be associated with poor prognosis among those with ASCVD, and a clinical trial population is likely under-representative of these higher risk, lower SES patients. Future studies are needed to test the simplified and extended risk scores in a more diverse cohort beyond clinical trial settings. Finally, validation of the extended model in the ACCORD trial was not possible as data on carotid artery stenosis, micro- versus macroalbuminuria, and COPD were not included in the trial report.

## Conclusion

In analyses from 14,671 individuals with T2DM and established ASCVD, a novel 4-year risk score was developed and validated to predict MACE. Using variables commonly available in the clinical setting, the risk score helps quantify the risk of developing future adverse CV events. The 9-factor risk score may identify individuals with the highest likelihood of developing MACE who may benefit from the most aggressive targeted secondary prevention therapies and warrant treatment with more expensive therapies given an increased cost efficiency of risk reduction.

## Supplementary Information

**Additional file 1. **Supplemental methods and tables.

## Data Availability

Requests to access the data for this study from qualified researchers trained in human subject confidentiality protocols may be submitted at dcri.org/data-sharing.

## Abbreviations

ACCORD: Action to Control Cardiovascular Risk in Diabetes
ASCVD: Atherosclerotic cardiovascular disease
CV: Cardiovascular
MACE: Major adverse cardiovascular events
MI: Myocardial infarction
T2DM: Type 2 diabetes mellitus
TECOS: Trial Evaluating Cardiovascular Outcomes with Sitagliptin

## References

[1] Rawshani A, Rawshani A, Franzen S, Sattar N, Eliasson B, Svensson AM (2018). Risk factors, mortality, and cardiovascular outcomes in patients with type 2 diabetes. N Engl J Med.

[2] Cavender MA, Steg PG, Smith SC, Eagle K, Ohman EM, Goto S (2015). Impact of diabetes mellitus on hospitalization for heart failure, cardiovascular events, and death: outcomes at 4 years from the Reduction of Atherothrombosis for Continued Health (REACH) Registry. Circulation.

[3] Rana JS, Liu JY, Moffet HH, Jaffe M, Karter AJ (2016). Diabetes and prior coronary heart disease are not necessarily risk equivalent for future coronary heart disease events. J Gen Intern Med.

[4] Rawshani A, Rawshani A, Franzen S, Eliasson B, Svensson AM, Miftaraj M (2017). Mortality and cardiovascular disease in type 1 and type 2 diabetes. N Engl J Med.

[5] Stevens RJ, Kothari V, Adler AI, Stratton IM, United Kingdom Prospective Diabetes Study Group (2001). The UKPDS risk engine: a model for the risk of coronary heart disease in Type II diabetes (UKPDS 56). Clin Sci (Lond).

[6] Fitchett D, Inzucchi SE, Cannon CP, McGuire DK, Scirica BM, Johansen OE (2019). Empagliflozin reduced mortality and hospitalization for heart failure across the spectrum of cardiovascular risk in the EMPA-REG OUTCOME Trial. Circulation.

[7] Hayes AJ, Leal J, Gray AM, Holman RR, Clarke PM (2013). UKPDS outcomes model 2: a new version of a model to simulate lifetime health outcomes of patients with type 2 diabetes mellitus using data from the 30 year United Kingdom Prospective Diabetes Study: UKPDS 82. Diabetologia.

[8] Plante TB, Juraschek SP, Zakai NA, Tracy RP, Cushman M (2019). Comparison of frequency of atherosclerotic cardiovascular disease events among primary and secondary prevention subgroups of the systolic blood pressure intervention trial. Am J Cardiol.

[9] Green JB, Bethel MA, Armstrong PW, Buse JB, Engel SS, Garg J (2015). Effect of Sitagliptin on cardiovascular outcomes in type 2 diabetes. N Engl J Med.

[10] Green JB, Bethel MA, Paul SK, Ring A, Kaufman KD, Shapiro DR (2013). Rationale, design, and organization of a randomized, controlled Trial Evaluating Cardiovascular Outcomes with Sitagliptin (TECOS) in patients with type 2 diabetes and established cardiovascular disease. Am Heart J.

[11] van Buuren S (2007). Multiple imputation of discrete and continuous data by fully conditional specification. Stat Methods Med Res.

[12] Harrell FE (2013). Regression modeling strategies: with applications to linear models, logistic regression, and survival analysis.

[13] Rubin DB (1987). Multiple imputation for nonresponse in surveys.

[14] Tibshurani R (1997). The LASSO Method for Variable Selection in the Cox Model. Stat Med.

[15] Harrell FE, Califf RM, Pryor DB, Lee KL, Rosati RA (1982). Evaluating the yield of medical tests. JAMA.

[16] D’Agostino RB, Nam B-H (2003). Evaluation of the performance of survival analysis models: discrimination and calibration measures.

[17] Buse JB, Bigger JT, Byington RP, Cooper LS, Cushman WC, ACCORD Study Group (2007). Action to Control Cardiovascular Risk in Diabetes (ACCORD) trial: design and methods. Am J Cardiol.

[18] Gerstein HC, Miller ME, Byington RP, Goff DC, Bigger JT, Action to Control Cardiovascular Risk in Diabetes Study Group (2008). Effects of intensive glucose lowering in type 2 diabetes. N Engl J Med.

[19] Zinman B, Wanner C, Lachin JM, Fitchett D, Bluhmki E, Hantel S (2015). Empagliflozin, cardiovascular outcomes, and mortality in type 2 diabetes. N Engl J Med.

[20] Wiviott SD, Raz I, Bonaca MP, Mosenzon O, Kato ET, Cahn A (2019). Dapagliflozin and cardiovascular outcomes in type 2 diabetes. N Engl J Med.

[21] Zelniker TA, Wiviott SD, Raz I, Im K, Goodrich EL, Bonaca MP (2019). SGLT2 inhibitors for primary and secondary prevention of cardiovascular and renal outcomes in type 2 diabetes: a systematic review and meta-analysis of cardiovascular outcome trials. Lancet.

[22] Zhuo X, Zhang P, Kahn HS, Bardenheier BH, Li R, Gregg EW (2015). Change in medical spending attributable to diabetes: national data from 1987 to 2011. Diabetes Care.

[23] Smith-Spangler CM, Bhattacharya J, Goldhaber-Fiebert JD (2012). Diabetes, its treatment, and catastrophic medical spending in 35 developing countries. Diabetes Care.

[24] Vaduganathan M, Sathiyakumar V, Singh A, McCarthy CP, Qamar A, Januzzi JL (2018). Prescriber patterns of SGLT2i after expansions of U.S. Food and Drug Administration labeling. J Am Coll Cardiol.

[25] Das SR, Everett BM, Birtcher KK, Brown JM, Januzzi JL, Kalyani RR (2020). 2020 expert consensus decision pathway on novel therapies for cardiovascular risk reduction in patients with type 2 diabetes: a report of the American College of Cardiology Solution Set Oversight Committee. J Am Coll Cardiol.

[26] Dorresteijn JA, Visseren FL, Wassink AM, Gondrie MJ, Steyerberg EW, Ridker PM (2013). Development and validation of a prediction rule for recurrent vascular events based on a cohort study of patients with arterial disease: the SMART risk score. Heart.

[27] National Cholesterol Education Program Expert Panel on Detection (2002). Evaluation, and Treatment of High Blood Cholesterol in Adults. Third Report of the National Cholesterol Education Program (NCEP) Expert Panel on Detection, Evaluation, and Treatment of High Blood Cholesterol in Adults (Adult Treatment Panel III) final report. Circulation.

[28] Anderson KM, Odell PM, Wilson PW, Kannel WB (1991). Cardiovascular disease risk profiles. Am Heart J.

[29] Anderson KM, Wilson PW, Odell PM, Kannel WB (1991). An updated coronary risk profile. A statement for health professionals. Circulation.

[30] Cederholm J, Eeg-Olofsson K, Eliasson B, Zethelius B, Nilsson PM, Gudbjornsdottir S, Swedish National Diabetes Register (2008). Risk prediction of cardiovascular disease in type 2 diabetes: a risk equation from the Swedish National Diabetes Register. Diabetes Care.

[31] Coleman RL, Stevens RJ, Retnakaran R, Holman RR (2007). Framingham, SCORE, and DECODE risk equations do not provide reliable cardiovascular risk estimates in type 2 diabetes. Diabetes Care.

[32] Kavaric N, Klisic A, Ninic A (2018). Cardiovascular risk estimated by UKPDS risk engine algorithm in diabetes. Open Med (Wars).

