# Supplementary material for: Development and validation of a model to predict cardiovascular death, nonfatal myocardial infarction, or nonfatal stroke in patients with type 2 diabetes mellitus and established atherosclerotic cardiovascular disease
Source: Cardiovasc Diabetol. 2022 Aug 27;21:166. doi: 10.1186/s12933-022-01603-8 (PMC9420281; doi:10.1186/s12933-022-01603-8)
Supplement: Supplementary file 1 — Additional file 1. Supplemental methods and tables. [file 12933_2022_1603_MOESM1_ESM.docx]

**SUPPLEMENTAL MATERIAL**

**SUPPLEMENTAL METHODS**

**Variables used in multiple imputation**

Age, sex, race, region, ethnicity, myocardial infarction, coronary occlusions ≥ 50%, CABG, PCI, smoking status, cerebrovascular disease, peripheral arterial disease, heart failure, NYHA class, chronic obstructive pulmonary disease, atrial fibrillation or atrial flutter, liver disease, depression, cancer within past 5 years, dyslipidemia, cholesterol, triglycerides, LDL, HDL, hemoglobin, HbA_1c_, diabetes duration, systolic blood pressure, diastolic blood pressure, heart rate, height, weight, eGFR, urinary albumin creatinine ratio, metformin, sulfonylurea, thiazolidinedione, insulin, beta blockers, ACE inhibitors, ARBs, calcium channel blockers, diuretics, aspirin, thienopyridine, statin, ezetimibe, VKA, niacin, fibrate, aldosterone antagonists, NSAIDs, digoxin, low molecular weight heparin, amputation, blindness, diabetic neuropathy, foot ulcers, and retinopathy.

**Detailed Statistical Analysis**

Two Cox proportional hazards regression models for MACE were developed. Candidates for the full model included demographic and medical history variables as well as lab values. The candidate set for a more parsimonious model that was specified to have no more than ten degrees of freedom was limited to demographic and medical history variables available at the bedside. Details related to the development of these models follow.

Twenty-five imputed datasets were created using SAS PROC MI with the method of fully conditional specification [11]. The list of variables used for imputation are provided above. Variables with more than 25% missing were imputed but were not candidates for the final model. Hazard ratios and p-values that are presented use Rubin’s method of multiple imputation [13] to combine the estimates from the 25 imputed datasets. For model calibration, and testing of modeling assumptions, only the first imputed dataset was used.

The linearity assumption of Cox regression was tested for each continuous variable and the estimated association with the hazard of MACE was plotted using the model with restricted cubic splines. In the case of non-linearity, a cut-point was chosen based on visual inspection of the plots and two linear splines were defined as candidates for model selection [12]. Tests of the proportional hazards assumption were done for all candidate variables. When a violation of the proportional hazards assumption was found, visual inspection of plots of scaled Schoenfeld residuals were used to determine whether violations would warrant concern. All pairwise Spearman correlation coefficients were calculated among candidate predictors. When correlations were greater than ρ>0.5, only one of the correlated variables was allowed to enter the model.

The full model was developed using stepwise variable selection with alpha=0.05 to enter and to stay in the model. After checking modeling assumptions, all variables shown in Table 1 were candidate variables for the full model except the following: urine albumin-to-creatinine ratio (UACR) and hemoglobin were not candidates because they had more than 25% missing. Weight was not a candidate because of collinearity with height and BMI. Region was not a candidate because of collinearity with race and ethnicity. Non-HDL cholesterol was pre-specified to be the most clinically relevant of the lipid measures and was used in place of individual variables for HDL, LDL, triglycerides, and total cholesterol. Insulin was the only medication that was a candidate for inclusion because of confounding with medical history variables. A single variable indicating whether any microvascular diabetic complication was present was a candidate in place of blindness, amputation, foot ulcer, diabetic neuropathy, and retinopathy. Two candidate variables were included for diastolic blood pressure (splines above and below 80 mmHg), heart rate (splines above and below 60 mmHg), body mass index (splines above and below 25 kg/m^2^), and eGFR (splines above and below 80 mL/min/1.73m^2^). Models were fit in each of the 25 datasets, and variables selected in at least 20 of the 25 datasets comprise the final models [13]. In the resulting model, all levels of categorical variables and both linear splines were included even when only some levels were selected.

A more parsimonious model was fit using Cox proportional hazards regression with least absolute shrinkage and selection operator (LASSO) methods [14]. For this method, the model was specified to have no more than 10 degrees of freedom, and only demographic and medical history variables available at the bedside were candidates. The adjusted R-square statistic was the criteria used for selection. For this model, chronic kidney disease was approximated with eGFR < 60 mL/min/1.73m^2^, so this model can be used without requiring recent lab results.

All variables that were considered for the predictive model are summarized overall and by whether the patient experienced MACE during the trial. Continuous variables are summarized with median (25^th^, 75^th^ percentile) and categorical variables with number and percentage. The numbers of nonmissing values before imputation are presented for each potential predictor. When relationships were found to be non-linear, the p-value corresponds to the model containing two piecewise linear splines.

**Table S1.** Number nonmissing for each candidate variable in the TECOS cohort

|  | **Overall (N=14,671)** | **No MACE (N=13,180)** | **MACE (N=1491)** |
| --- | --- | --- | --- |
| Age | 14,351 | 12,920 | 1431 |
| Female | 14,671 | 13,180 | 1491 |
| Race | 14,671 | 13,180 | 1491 |
| Hispanic ethnicity | 14,671 | 13,180 | 1491 |
| Region | 14,671 | 13,180 | 1491 |
| Duration of type 2 diabetes | 14,659 | 13,171 | 1488 |
| HbA1c | 14,666 | 13,176 | 1490 |
| Height | 14,545 | 13,074 | 1471 |
| Weight | 14,599 | 13,121 | 1478 |
| Body mass index | 14,534 | 13,067 | 1467 |
| Systolic blood pressure | 14,629 | 13,142 | 1487 |
| Diastolic blood pressure | 14,629 | 13,142 | 1487 |
| Heart rate | 14,460 | 12,983 | 1477 |
| eGFR | 14,528 | 13,056 | 1472 |
| CKD (eGFR<60) | 14,528 | 13,056 | 1472 |
| UACR | 5,088 | 4,567 | 521 |
| Hemoglobin | 9,568 | 8,592 | 976 |
| Non-HDL-c | 11,646 | 10,482 | 1164 |
| HDL-c | 11,760 | 10,586 | 1174 |
| LDL-c | 11,066 | 9,966 | 1100 |
| Triglycerides | 12,189 | 10,988 | 1201 |
| Prior myocardial infarction | 14,671 | 13,180 | 1491 |
| ≥ 50% coronary stenosis | 14,671 | 13,180 | 1491 |
| Prior PCI | 14,468 | 12,996 | 1472 |
| Prior CABG | 14,671 | 13,180 | 1491 |
| Prior stroke | 14,671 | 13,180 | 1491 |
| Prior TIA | 14,671 | 13,180 | 1491 |
| ≥ 50% stenosis in the carotid artery | 14,671 | 13,180 | 1491 |
| Peripheral arterial disease | 14,671 | 13,180 | 1491 |
| NYHA class | 14,248 | 12,830 | 1418 |
| Cigarette smoking status | 14,671 | 13,180 | 1491 |
| Hypertension | 14,671 | 13,180 | 1491 |
| Dyslipidemia | 14,671 | 13,180 | 1491 |
| COPD | 14,671 | 13,180 | 1491 |
| Atrial fibrillation/flutter | 14,671 | 13,180 | 1491 |
| Cancer within the past 5 years | 14,671 | 13,180 | 1491 |
| Depression | 14,671 | 13,180 | 1491 |
| Liver disease | 14,671 | 13,180 | 1491 |
| Any microvascular complication | 14,671 | 13,180 | 1491 |
| Blindness | 14,671 | 13,180 | 1491 |
| Retinopathy | 14,670 | 13,179 | 1491 |
| Amputation | 14,671 | 13,180 | 1491 |
| Diabetic neuropathy | 14,671 | 13,180 | 1491 |
| Foot ulcers | 14,671 | 13,180 | 1491 |
| Albuminuria | 11,635 | 10,498 | 1137 |
| Insulin | 14,671 | 13,180 | 1491 |
| Sulfonylurea | 14,671 | 13,180 | 1491 |
| Metformin | 14,671 | 13,180 | 1491 |
| ACE inhibitor or ARB | 14,671 | 13,180 | 1491 |
| Beta blocker | 14,671 | 13,180 | 1491 |
| Calcium channel blocker | 14,671 | 13,180 | 1491 |
| Diuretic | 14,671 | 13,180 | 1491 |
| Aldosterone antagonist | 14,671 | 13,180 | 1491 |
| Aspirin | 14,671 | 13,180 | 1491 |
| Thienopyridine | 14,671 | 13,180 | 1491 |
| VKA | 14,671 | 13,180 | 1491 |
| Fibrate | 14,671 | 13,180 | 1491 |
| NSAIDs | 14,671 | 13,180 | 1491 |
| Statin | 14,671 | 13,180 | 1491 |
| Ezetimibe | 14,671 | 13,180 | 1491 |
| Fibrate | 14,671 | 13,180 | 1491 |

ACE, angiotensin-converting enzyme; ARB, angiotensin receptor blocker; CABG, coronary artery bypass graft; CKD, chronic kidney disease; COPD, chronic obstructive pulmonary disease; eGFR, estimated glomerular filtration rate, HDL-c, high-density lipoprotein cholesterol; LDL-c, HDL-c, low-density lipoprotein cholesterol; MACE, major adverse cardiac event; NSAIDs, nonsteroidal anti-inflammatory drugs; NYHA, New York Heart Association; PCI, percutaneous coronary intervention; TECOS, Trial Evaluating Cardiovascular Outcomes with Sitagliptin; TIA, transient ischemic attack; UACR, urine albumin-to-creatinine ratio; VKA, vitamin K antagonist.

**Table S2: Multivariable adjusted model output parameters using variables in the extended risk score for cardiovascular death, nonfatal myocardial infarction, or nonfatal ischemic stroke**

| **Variable** | **Parameter Estimate** | **HR (95% CI)** | **p** |
| --- | --- | --- | --- |
| Age, per 10-year increase | 0.314755 | 1.37 (1.27, 1.47) | <0.0001 |
| Stroke | 0.516589 | 1.68 (1.47, 1.91) | <0.0001 |
| Myocardial infarction | 0.387038 | 1.47 (1.32, 1.64) | <0.0001 |
| eGFR |  |  | <0.0001 |
| HR for 10 unit increase to 80 mL/min/1.73m^2^ | -0.150827 | 0.86 (0.82, 0.90) |  |
| HR for 10 unit increase above 80 mL/min/1.73m^2^ | 0.070815 | 1.07 (1.02, 1.13) |  |
| Male | 0.335020 | 1.40 (1.23, 1.59) | <0.0001 |
| Non-HDL-c, HR for 10-unit increase in mg/dL | 0.032854 | 1.03 (1.02, 1.05) | <0.0001 |
| NYHA Class (No CHF is reference) |  |  | <0.0001 |
| I | 0.161444 | 1.18 (0.92, 1.50) |  |
| II | 0.351994 | 1.42 (1.22, 1.66) |  |
| III | 0.405745 | 1.50 (1.16, 1.94) |  |
| IV | 1.481878 | 4.40 (1.90, 10.19) |  |
| Insulin use | 0.288882 | 1.33 (1.18, 1.51) | <0.0001 |
| Heart rate |  |  | <0.0001 |
| HR for 10 bpm increase to 60 | -0.384747 | 0.68 (0.54, 0.86) |  |
| HR for 10 bpm increase above 60 | 0.107113 | 1.11 (1.05, 1.18) |  |
| Diastolic blood pressure |  |  | 0.0002 |
| HR for 10 mmHg increase to 80 | -0.133314 | 0.88 (0.81, 0.94) |  |
| HR for 10 mmHg increase above 80 | 0.174872 | 1.19 (1.07, 1.32) |  |
| Albuminuria (reference is none) |  |  | 0.0002 |
| Microalbuminuria | 0.255011 | 1.29 (1.11, 1.49) |  |
| Macroalbuminuria | 0.352949 | 1.42 (1.11, 1.83) |  |
| Atrial flutter or fibrillation | 0.296578 | 1.35 (1.15, 1.58) | 0.0002 |
| Body mass index |  |  | 0.0004 |
| HR for 1 kg/m^2^ increase to 25 kg/m^2^ | -0.101855 | 0.90 (0.86, 0.95) |  |
| HR for 1 kg/m^2^ increase above 25 kg/m^2^ | 0.005405 | 1.01 (0.99, 1.02) |  |
| Coronary artery disease | 0.206912 | 1.23 (1.10, 1.38) | 0.0004 |
| Smoking (reference is never) |  |  | 0.0010 |
| Current | 0.292440 | 1.34 (1.13, 1.59) |  |
| Former | -0.006959 | 0.99 (0.88, 1.12) |  |
| Any diabetes-specific microvascular comorbidity (blindness, amputation, foot ulcer, diabetic neuropathy, or retinopathy) | 0.139161 | 1.15 (1.03, 1.29) | 0.0158 |
| Dyslipidemia | -0.149648 | 0.86 (0.76, 0.98) | 0.0206 |
| ≥ 50% stenosis of carotid artery | 0.223347 | 1.25 (1.02, 1.53) | 0.0279 |
| COPD | 0.183735 | 1.20 (1.01, 1.43) | 0.0348 |

COPD, chronic obstructive pulmonary disorder; eGFR, estimated glomerular filtration rate; HDL-c, high-density lipoprotein cholesterol; NYHA, New York Heart Association.

* The baseline survival function is 0.4270 at 1 year, 0.1913 at 2 years, 0.0822 at 3 years, and 0.0358 at 4 years; C-index: 0.678 (0.664, 0.692)

**Table S3: Multivariable adjusted model output parameters using variables in the parsimonious risk score for cardiovascular death, nonfatal myocardial infarction, or nonfatal ischemic stroke**

| **Variable** | **Parameter Estimate^*^** | **HR (95% CI)** | **p** |
| --- | --- | --- | --- |
| Age (per 10-year increase) | 0.278594 | 1.32 (1.23, 1.41) | <0.0001 |
| Prior stroke | 0.503503 | 1.65 (1.46, 1.87) | <0.0001 |
| Prior myocardial infarction | 0.381841 | 1.46 (1.31, 1.63) | <0.0001 |
| Chronic kidney disease | 0.396991 | 1.49 (1.33, 1.67) | <0.0001 |
| Heart failure | 0.343585 | 1.41 (1.25, 1.59) | <0.0001 |
| Male | 0.320449 | 1.38 (1.22, 1.55) | <0.0001 |
| Insulin use | 0.305388 | 1.36 (1.20, 1.53) | <0.0001 |
| Atrial fibrillation or flutter | 0.337737 | 1.40 (1.20, 1.64) | <0.0001 |
| Any diabetes-specific microvascular comorbidity (blindness, amputation, foot ulcer, diabetic neuropathy, or retinopathy) | 0.191871 | 1.21 (1.09, 1.35) | 0.0006 |

* The baseline survival function is 0.9976 at 1 year, 0.9954 at 2 years, 0.9930 at 3 years, and 0.9906 at 4 years; C-index: 0.649 (0.635, 0.664)

**Table S4.** Baseline characteristics of the external validation ACCORD cohort.

|  | **N non-missing** | **Overall** | **No MACE Event** | **MACE Event** |
| --- | --- | --- | --- | --- |
| n | 1404 | 1404 | 1274 | 130 |
| Age, y | 1404 | 64.0 (58.5, 69.5) | 63.8 (58.3, 69.3) | 65.5 (60.1, 70.3) |
| eGFR, mL/min/1.73m^2^ | 1404 | 85.8 (71.0, 102.4) | 85.2 (71.2, 102.5) | 87.2 (70.0, 99.1) |
| History of myocardial infarction | 1404 | 777 (55.3) | 710 (55.7) | 67 (51.5) |
| Female | 1404 | 360 (25.6) | 328 (25.7) | 32 (24.6) |
| History of heart failure | 1403 | 1268 (90.4) | 1161 (91.2) | 107 (82.3) |
| History of atrial fibrillation | 1400 | 19 (1.4) | 17 (1.3) | 2 (1.5) |
| HDL-c, mg/dL | 1404 | 38.0 (33.0, 45.0) | 38.0 (33.0, 45.0) | 38.0 (32.0, 44.0) |
| Total cholesterol, mg/dL | 1403 | 167.0 (146.0, 193.0) | 167.0 (146.0, 193.0) | 173.0 (146.2, 198.0) |
| Diastolic blood pressure, mmHg | 1400 | 71.0 (64.0, 79.0) | 72.0 (65.0, 79.0) | 69.0 (63.0, 76.0) |
| Heart rate, bpm | 1399 | 68.0 (60.0, 75.5) | 68.0 (60.0, 75.0) | 67.0 (59.0, 76.0) |
| Micro or macroalbuminuria | 1404 | 343 (24.4) | 300 (23.5) | 43 (33.1) |
| Body mass index, kg/m^2^ | 1402 | 31.1 (28.0, 35.2) | 31.2 (28.1, 35.2) | 30.5 (27.5, 34.8) |
| History of CABG | 1404 | 940 (67.0) | 859 (67.4) | 81 (62.3) |
| History of angina | 1404 | 938 (66.8) | 846 (66.4) | 92 (70.8) |
| Carotid stenosis | 1404 | 138 (9.8) | 127 (10.0) | 11 (8.5) |
| Years of hyperlipidemia | 1083 | 6.0 (3.0, 10.0) | 5.5 (3.0, 10.0) | 6.0 (3.0, 12.0) |
| Asthma | 1401 | 39 (2.8) | 36 (2.8) | 3 (2.3) |
| Hemoglobin A1c, % | 1404 | 7.5 (7.2, 7.7) | 7.5 (7.2, 7.7) | 7.5 (7.3, 7.8) |
| Insulin use | 1403 | 497 (35.4) | 452 (35.5) | 45 (34.6) |
| Microvascular complications | 1302 | 518 (39.8) | 451 (38.3) | 67 (54.0) |
| History of CKD | 1404 | 150 (10.7) | 136 (10.7) | 14 (10.8) |

Data shown are median (25th, 75th percentile) or n (%). ACCORD, Action to Control Cardiovascular Risk in Diabetes; CABG, coronary artery bypass graft; CKD, chronic kidney disease; eGFR, estimated glomerular filtration rate, HDL-c, high-density lipoprotein cholesterol; MACE, major adverse cardiac event.
